# The (cost-)effectiveness of a lifestyle physical activity intervention in addition to a work style intervention on the recovery from neck and upper limb symptoms in computer workers

**DOI:** 10.1186/1471-2474-7-80

**Published:** 2006-10-24

**Authors:** Claire M Bernaards, Geertje AM Ariëns, Vincent H Hildebrandt

**Affiliations:** 1 Body@Work Research Center Physical Activity, Work and Health, TNO-VUmc, Amsterdam, The Netherlands; 2 Institute for Research in Extramural Medicine, Department of Occupational and Public Health, VU University Medical Center, Amsterdam, The Netherlands; 3 TNO Quality of Life, Leiden, The Netherlands; 4 Municipal Health Service The Hague, The Netherlands

## Abstract

**Background:**

Neck and upper limb symptoms are frequently reported by computer workers. Work style interventions are most commonly used to reduce work-related neck and upper limb symptoms but lifestyle physical activity interventions are becoming more popular to enhance workers health and reduce work-related symptoms. A combined approach targeting work style and lifestyle physical activity seems promising, but little is known on the effectiveness of such combined interventions.

**Methods/design:**

The RSI@Work study is a randomised controlled trial that aims to assess the added value of a lifestyle physical activity intervention in addition to a work style intervention to reduce neck and upper limb symptoms in computer workers. Computer workers from seven Dutch companies with frequent or long-term neck and upper limb symptoms in the preceding six months and/or the last two weeks are randomised into three groups: (1) work style group, (2) work style and physical activity group, or (3) control group. The work style intervention consists of six group meetings in a six month period that take place at the workplace, during work time, and under the supervision of a specially trained counsellor. The goal of this intervention is to stimulate workplace adjustment and to improve body posture, the number and quality of breaks and coping behaviour with regard to high work demands. In the combined (work style and physical activity) intervention the additional goal is to increase moderate to heavy physical activity. The control group receives usual care. Primary outcome measures are degree of recovery, pain intensity, disability, number of days with neck and upper limb symptoms, and number of months without neck and upper limb symptoms. Outcome measures will be assessed at baseline and six and 12 months after randomisation. Cost-effectiveness of the group meetings will be assessed using an employer's perspective.

**Discussion:**

This study will be one of the first to assess the added value of a lifestyle physical activity intervention in addition to a work style intervention in reducing neck and upper limb symptoms of computer workers. The results of the study are expected in 2007.

## Background

### Introduction

Neck and upper limb symptoms are frequently reported by computer workers. More than 50% of the computer workers report symptoms in neck, shoulders, arms, wrists or fingers [1]. In the year 2002, 28% of the general Dutch working population suffered from pain or stiffness in the neck, shoulder, arms, hands or wrists in the previous 12 months [2]. In Europe the prevalence for work-related neck/shoulder pain was 25% and 15% for work-related arm pain [3]. The total yearly costs of neck and upper limb symptoms in the Netherlands due to decreased productivity, sick leave, chronic disability for work and medical costs were recently estimated at 2.1 billion euros [4].

Several names have been used to indicate symptoms in neck, shoulders, arms, hands or wrists. The most commonly used names are cumulative trauma disorders (CTDs), musculoskeletal disorders (MSDs), upper limb disorders (ULDs), upper extremity disorders (UEDs) and repetitive strain injury (RSI). In October 2004 the name CANS has been introduced in the Netherlands to indicate complaints in arm, neck and shoulder. However, since the general Dutch population is more familiar with the name RSI, the name of the present study is RSI@Work study. This study deals with non-specific work-related neck and upper limb symptoms.

### Rationale behind the study

The general model behind the present study is the dynamic model of workload presented by Van Dijk et al. [5]. According to this model, health effects may result from an imbalance between workload and capacity. By increasing capacity and decreasing workload this imbalance may be restored. The present study is designed to assess the effectiveness of an intervention that intends to decrease workload by improving work style and to increase capacity by improving general physical activity of computer workers with neck and upper limb symptoms. By comparing the effectiveness of a combined intervention (work style and physical activity) with a single intervention (work style) and a control intervention (usual care), the added value of a lifestyle physical activity intervention in addition to a work style intervention will be assessed. In addition, the effectiveness of a combined and single intervention will be compared with the effectiveness of usual care. Although general capacity is the result of physical as well as mental capacity, the RSI@Work study will primarily focus on the physical component.

In order to reduce workload, we will use a multidisciplinary approach to improve work style. The concept of work style was constructed by Feuerstein et al. [6] and represents the individual responses to high work demands. In the present study we defined work style as the following three individual responses to high work demands: 1. optimal body posture and proper workplace adjustment, 2. the number and quality of breaks and 3. coping with high work demands. The general idea is that, in case of high work demands, workers will be less inclined to pay attention to their body posture, adjust their workplace properly and use break and exercise reminder software.

In order to improve physical capacity, workers are stimulated to increase their general physical activity by means of active commuting, sports, and daily physical activities at work and/or during leisure time. Increasing physical activity might be effective in reducing neck and upper limb symptoms through three different mechanisms. The first mechanism is based on the improvement of muscle functioning. In a study with neck and shoulder myalgia, Kadi et al. [7] showed an increase in oxidative capacity (i.e. capillary density) and muscle functioning of the trapezius muscle resulting from strength and endurance training. High oxidative capacity may prevent high calcium levels and mitonchondrial damage resulting from continuous activation of type 1 muscles during computer work. The second mechanism is based on a discontinuation of repetitive movements during computer work by physical activity at the workplace. Byl et al. [8] showed changes in the central nervous system and motor deterioration in owl monkeys performing "highly articulated" repetitive movements in precision tasks. Changes in the central nervous system as described by Byl et al. [8] may underlie neck and upper limb symptoms in computer workers. The third mechanism is based on the role of physical activity in coping with high work demands and stress. Physical activity stimulates muscle relaxation after physical exertion and can distract workers from stressful work aspects. Furthermore, physical activity may reduce stress through increased self-efficacy and self-esteem [9].

Several studies have been published on the effectiveness of exercise programs to reduce pain and disability in patients with neck and shoulder symptoms [10-22]. Many of these studies showed positive effects on the reduction of neck and/or shoulder symptoms [10,14,16,17,19-22] but not all [11-13,15,17,18]. However, most of these studies show only short-term effects of exercise programs [10,14,17,19,21]. Long-term adherence to exercise programs is often low, possibly due to lack of pleasure or high barriers to visit a health club [20]. Pleasure seems to be an important predictor of walking, moderate intensity and strenuous intensity physical activity [23]. Lifestyle physical activity interventions that aim to increase physical activity up to at least 30 minutes per day seem to be promising with regard to the maintenance of physical activity [24]. In contrast to exercise programs, the primary goal of lifestyle physical activity interventions is to integrate moderate intensity physical activities into daily life [24]. A key feature of lifestyle physical activity interventions is that physical activities are not prescribed but chosen by the participants.

## Methods/design

### Design and study population

The RSI@Work study is a Randomised Controlled Trial (RCT) with two intervention groups and a control group, an intervention period of six month and measurements at baseline and after six and 12 months of follow-up. Computer workers (N = ± 8000) from the head-offices of 7 Dutch companies in various branches (e.g. insurance, science, energy, transportation-policy and taxes) in different regions of the Netherlands received a short questionnaire about symptoms (i.e. pain, stiffness, tingles) in neck, shoulders, arms, wrists and hands in the past two weeks and the past six months. Furthermore they received written information about the study, and a letter in which they were requested to fill out the questionnaire and send it to the researchers. At the end of the short questionnaire, workers could indicate whether or not they were willing to participate in the study. The researchers used the short questionnaire to select workers who were eligible for participation in the study. The following inclusion criteria were used:

1) Frequent (i.e. at least once a week) or long term pain, stiffness or tingles in neck, shoulders, arms, wrists and/or hands in the preceding six months and/or the last two weeks. By using a six-month period and a two-week period, we intend to include workers with chronic and recurrent symptoms as well as workers with recent symptoms.

2) Performing computer work for at least three days a week during at least three hours a day.

3) A working contract until the last follow-up measurement.

4) Not under treatment of a doctor or (physical) therapist for complaints in the neck, shoulders arms, wrists and/or hands.

5) No non-work related or clear somatic diseases (e.g. rheumatoid arthritis, cervical hernia, tennis elbow, carpal tunnel syndrome).

6) Sickness absence of less than 50%.

Women who were pregnant at the start of the study were excluded since they were unable to complete the study due to their pregnancy leave. Workers who met all inclusion criteria and were willing to participate in the study received a written informed consent, a baseline questionnaire, a short medical questionnaire and an invitation to participate in the baseline measurements. The baseline measurements consisted off a submaximal cardiorespiratory fitness test, a measurement of body height and body weight, a maximum grip strength test and a workplace observation. The baseline measurements were conducted within one month after receiving the baseline questionnaire. At the day of the baseline measurements the workers handed over their completed questionnaire and written informed consent to the researchers. Workers who were unable to participate in the baseline measurements were asked to send their completed baseline questionnaire and informed consent to the VU university medical center. All workers who gave informed consent and completed the baseline questionnaire were randomised into one of the two intervention groups (1. the work style group, 2. the work style plus physical activity group) or the control group.

The study was approved by the Medical Ethics Committee of the VU University Medical Center (number 04/027) and all participants provided written informed consent. In May/June 2003 a pilot study was performed in three companies. The goal of this pilot study was to practice, evaluate and adjust the RSI@Work intervention. On the basis of the results of this pilot, small improvements in the design have been made to ensure the feasibility of the study in companies. The baseline measurements and follow-up measurements were conducted in October 2004 (T_0_), April 2005 (T_1_) and October 2005 (T_2_). The results of the RSI@Work study are expected in 2007.

### Treatment allocation

An independent statistician prepared the randomisation by using a computer-generated randomisation. To prevent unequal randomisation, workers were pre-stratified by company and baseline sports participation assessed with the baseline questionnaire. Furthermore, block randomisation with blocks of three was used. The researchers prepared the envelopes containing the allocation to the intervention group and made two boxes with envelopes for each company. The first box contained the envelopes for workers who participated in sports activities (i.e. at least twice a week during at least 20 minutes at a moderate intensity level). The second box contained the envelopes for workers who did not participate in sports activities. Directly after the baseline measurements were completed the researchers opened the envelope and informed the worker about the group allocation. Workers who did not participate in the baseline measurements but sent their completed questionnaire and informed consent by post were randomised at the VU university medical center and informed about their treatment allocation by phone or by email.

### Sample size and participant flow

**Figure 1:**
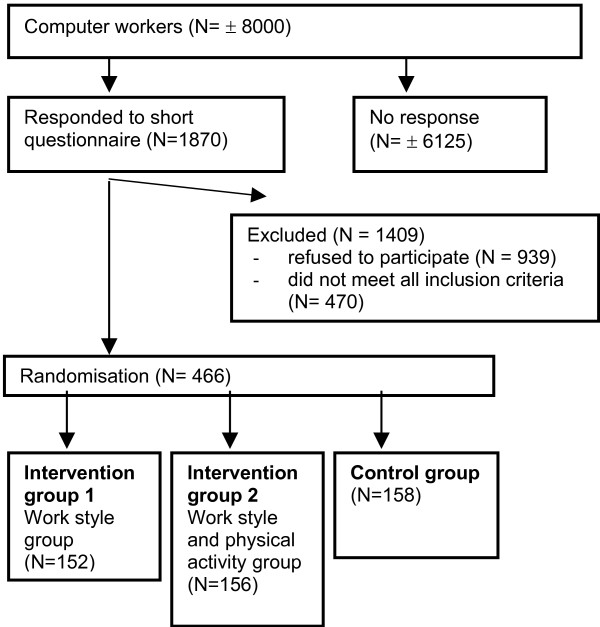
Participant flow chart.

The main primary outcome variable in this study is self-reported recovery. The intervention will be considered successful if recovery from work-related neck and upper limb symptoms (i.e. completely recovered or much recovered) is 20% higher in the intervention group than in the control group. The number of participants in each group needed to detect a difference in recovery of 20% between the intervention group and the control group was ± 80, with α = 0.05 (two sided) and β = 0.20. This number was based on our expectation that recovery would be 80% in the intervention group and 60% in the control group. However, since we expected a loss-to-follow-up of 40%, ± 135 workers were needed in each group at baseline. Based on our pilot study we expected that 6.25% of our source population would participate in the study, which brings the minimum number of workers that needed to be invited for participation at 6480 workers. Figure 1 shows that approximately 8000 workers were invited to participate and that 466 workers were randomised into the trial.

### Blinding

It was not possible to blind participants and counsellors for the treatment allocation. Participants in the intervention groups attended group meetings with other participants from the same intervention group whereas participants in the control group did not. Since the primary outcome variables were self-reported, blinding was impossible. However, the researchers who performed the measurements were not aware of the treatment allocation of participants except for the counsellors who also performed part of the measurements.

### Description of the intervention

Like previous lifestyle physical activity intervention studies [24], the RSI@Work intervention is based on theoretical models of behaviour change, i.e. the Trans Theoretical Model (TTM) and the Precaution Adoption Process Model (PAPM). The TTM separates behaviour change into five discrete stages that are defined by a person's past behaviour and his or her plans for future action [25]. The PAPM resembles the TTM stages but introduces two new stages. These new stages distinguish between people who are unaware of an issue (Stage 1) and those who know something about an issue but never actively thought about it (Stage 2) [26]. Concepts of the PAPM and TTM, such as stage of change, awareness, self-efficacy and decisional balance, are used in the group meetings and applied to both the work style and the combined (work style and physical activity) intervention.

The interventions for the two intervention groups both consist of six interactive group meetings in a six-month period. All group meetings take place at the workplace, during work time (with permission of the employee) and under the supervision of a specially trained counsellor. The counsellors use standardized protocols that have been tested and improved during the pilot study. Four out of six meetings are large group meetings with maximally 10 participants. Two out of six meetings are small group meetings with maximally four participants. The goal of all group meetings is behavioural change with regard to physical activity and/or work style. The goal of the large group meetings is to provide general information and to raise awareness about work style and/or physical activity, and to discuss and find solutions for general barriers with regard to behavioural change. The goal of the small group meetings is to provide participants with tailored advice based on their stage of change with regard to work style and/or physical activity. In addition, solutions for individual barriers with regard to behavioural change are discussed.

The counsellor provides the work style part to both intervention groups and the physical activity part to the 'work style and physical activity group' only. The group meetings of the 'work style and physical activity group' are separate from the group meetings of the 'work style group'.

#### Group meeting 1 (large group meeting)

*Work style *(1 hour). The goal is to provide general information about neck and upper limb symptoms and its known risk factors. The following risk factors are discussed: workplace adjustment and body posture, static workload and insufficient breaks, high workload and work stress. Finally, information is provided about the study and the next group meetings. *Physical activity *(30 minutes). The goal is to provide information about the importance of physical activity in the prevention or reduction of neck and upper limb symptoms and to raise awareness about participants' own physical activity pattern. The dynamic model of workload is presented to explain the role of physical inactivity in the development of neck and upper limb symptoms. The counsellor explains the Dutch guidelines (similar to ACSM/CDC guidelines) for regular physical activity (i.e. engaging in moderate intensity physical activity for at least 30 minutes on at least five days of the week) and strenuous physical activity (i.e. engaging in strenuous intensity physical activity for at least 20 minutes on at least three days of the week). Participants are requested to check whether or not their own daily physical activity pattern meets these guidelines. The counsellor stresses the importance of active commuting and sufficient physical activity at work and during leisure time. Participants are asked to make a physical activity plan, to act according to this plan and to write down perceived barriers that prevented them from being physically active as planned. Participants are also asked to think about barriers that might prevent them from being sufficiently physical active in the future. Workers who are sufficiently active at the start of the study according to the general guidelines are encouraged to improve their physical activities by spending more time on activities that involve the upper extremities or by becoming more active at work.

#### Group meeting 2 (large group meeting)

*Work style *(1 hour). The primary goal is to provide the general guidelines for workplace adjustment and body posture (Table 1) and to make participants aware of their own workplace and body posture. The second goal is to provide information about the importance of breaks and exercise reminder software and to stimulate the use of it. All participants get access to this software. *Physical activity *(30 minutes). The goal is to discuss the physical activity plans and the perceived and expected barriers. Groups are stimulated to find solutions for reported barriers. Participants who already meet the physical activity guidelines at baseline are encouraged to explain to others how they manage to be sufficiently active and cope with barriers. Participants who fail to perform their planned activities are asked to adjust their physical activity plan in order to make it more feasible. At the end of the meeting all participants receive an elastic band with exercises for the upper extremities.

#### Group meeting 3 (small group meeting)

*Work style *(30 minutes). The goal of this meeting is to bring the guidelines for workplace adjustment and body posture into practice. In a small group, participants visit each other's workplace. The counsellor will ask participants to judge and adjust each workplace according to the guidelines (Table 1). If necessary, the counsellor makes some final adjustments to the workplace at the end of the meeting. Furthermore, the use of break and exercise reminder software is discussed. The counsellor checks with the participants whether the software has been installed properly, whether the software has been used and discusses the experiences. The counsellors advice will be based on the stage of change of participants. If the workplace has not been adjusted or the software has not been used, barriers will be discussed as well as the importance of workplace adjustment and the use of breaks and exercise reminder software. If the workplace has been adjusted and software has been used, the counsellor encourages this behaviour and will ask participants about the use of the exercises that are provided by the exercise reminder software. *Physical activity *(15 minutes). If participants have not decided to change their physical activity behaviour, the counsellor will raise awareness and stress the positive aspects of physical activity and the risk of physical inactivity. If participants have decided to change their physical activity behaviour but have not started yet, the counsellor will discuss (individual) barriers together with the other participants. Furthermore, the counsellor will encourage participants to adjust their physical activity plan in order to make it more feasible. If participants started to become more physically active the counsellor will encourage this behaviour and discuss possible future barriers.

#### Group meeting 4 (large group meeting)

*Work style *(1 hour). The first goal is to provide information about high workload and work stress and the relationship with neck and upper limb symptoms. The second goal is to teach participants how to recognize work stress. The counsellor discusses biological (e.g. insomnia, headache, high blood pressure), behavioural (e.g. excessive smoking or drinking behaviour, restlessness) and emotional indicators of work stress (e.g. concentration difficulties, feelings of insecurity or guild, amnesia). The third goal is to raise awareness about general and individual risk factors for work stress during a brainstorm session. The counsellor provides each participant with three to five post-it stickers and asks them to write down a factor on each post-it sticker that he or she associated with work stress (e.g. too much work, unclear task descriptions). The most frequently reported risk factors are discussed. *Physical activity *(30 minutes). The goal is to discuss the physical activity plans and the experienced and expected barriers. Furthermore, the counsellor discusses the process of behavioural change.

#### Group meeting 5 (small group meeting)

*Work style *(30 minutes). The goal of this meeting is to discuss individual risk factors for work stress raised by participants. The group is stimulated to find solutions for risk factors and to discuss ways of coping with work stress as well as barriers to cope with work stress. In addition, the counsellor discusses the use of breaks and exercise reminder software, body posture and workplace adjustment. *Physical activity *(15 minutes). The goal is to evaluate participant's physical activity pattern and to give supplementary advice if necessary.

#### Group meeting 6 (large group meeting)

The goal is to summarize and evaluate all group meetings and to discuss how to attenuate behavioural changes.

Participants in the control group do not attend any of the group meetings. If they visit their occupational physician for neck and upper limb symptoms they receive usual care.

### Co-intervention and compliance (dropout)

Although all workers who reported to be under treatment of a doctor or (physical) therapist for complaints in the neck, shoulders arms, wrists and/or hands at the start of the study were excluded for participation, we allowed participants to visit a doctor or (physical) therapist during the study. In order to adjust for co-interventions participants were asked to report all actions taken to reduce their neck and upper limb symptoms (e.g. visiting a doctor or taking medication). Two estimates of compliance are used in our study, i.e. 1. compliance to the group meetings and 2. compliance to the study. During each group meeting the counsellor checks the presence of each participant.

### Contrast between intervention and usual care

In May 2003 the Dutch guidelines for the occupational health management of workers with complaints in arm, shoulder and neck were published [27]. According to these guidelines the occupational physician should advice workers with neck and upper limb symptoms to keep working but to discontinue tasks that cause intense pain. Furthermore, therapies that activate workers are preferred above usual physiotherapy. In case of bad workplace ergonomics, the guidelines recommend to improve the workplace in combination with personalized interventions. In case of stress, the guidelines recommend changes in work organization and work situations. If the worker has non-realistic cognitions the guidelines recommend the occupational physician to explain the multifactorial origin of arm, shoulder and neck symptoms and its good prognosis. If necessary, workers can be send to a psychologist. In case of sickness absence, workers will be stimulated to resume their work gradually based on a time contingent schedule of which the length is dependent on the seriousness of the complaints and the workload.

Although the RSI@Work intervention is based on the Dutch guidelines for the occupational health management of workers with complaints in arm, shoulder and neck, it differs from the guidelines with regard to three aspects. First of all, many computer workers with neck and upper limb symptoms who are not on sick leave do not visit their occupational physician. At baseline only 13.5% of the RSI@Work participants had visited their occupational physician for neck and upper limb symptoms. As a consequence many participants in the control group of this study will not receive the treatment as described by the guidelines for occupational health management. The participants in the intervention groups, on the other hand, will all be invited to attend the group meetings. Secondly, RSI@Work participants are not treated by a doctor or therapist but are guided by a counsellor. During the group meetings, the focus is on behavioural change instead of on symptoms. Participants of the RSI@Work study are offered knowledge and methods to reduce their neck and upper limb symptoms by themselves. For instance, they learn to adjust their own workplace by themselves which is in contrast to general occupational health management, where workplaces are often adjusted by occupational therapists. Although some companies provide workers with information about the adjustment of their workplace, workers often feel uncertain about changing their workplace by themselves. In addition, participants in the RSI@Work intervention groups are stimulated and guided in using breaks and exercise reminder software. In some Dutch companies breaks and exercise reminder software is available but guidance is often lacking. Furthermore, participants in the combined (work style and physical activity) intervention are stimulated to increase their physical activity, not by offering a training protocol but by helping them to find ways to incorporate physical activity into their daily lives. Although some large companies offer fitness facilities, this form of physical activity is not attractive for all workers. Training protocols and physical activities that do not fit the worker are believed not to be effective on the long-term. The third important difference between the RSI@Work intervention and usual occupational health management is the fact that the RSI@Work intervention consists of six group meetings. This provides the opportunity for feedback and to discuss positive and negative experiences with the new learned behaviours. In general, usual occupational health management is based on individual advice and treatment.

### Measurements

All outcome variables, except for degree of recovery and body height, will be assessed three times: prior to the start of the intervention (T0), at the end of the six month intervention (T1) and 12 months after the start of the intervention (T2). Degree of recovery is assessed at T1 and T2 and body height is measured at baseline only.

#### Primary outcome variables

1. Degree of recovery from neck and upper limb symptoms assessed using a 7-point scale ranging from "much worse" to "completely recovered" compared to baseline.

2. Pain intensity (current pain, average pain and worst pain in the past four weeks), assessed using an 11-point numerical rating scale ranging from 0 "no pain" to 10 "worst pain ever" [28].

3. Disability assessed using an 11-point numerical rating scale [28]. Change in ability to work in the past four weeks is assessed with Von Korff scales ranging from 0 "no change" to 10 "extreme change". Interference of pain on daily activities in the past four weeks is assessed with Von Korff scales ranging from 0 "no interference" to 10 "unable to carry on any activities".

4. The Dutch musculoskeletal questionnaire [29] is used to assess the six month prevalence of symptoms (pain, stiffness, tingles or numbness) in the neck, right shoulder, left shoulder, right arm, left arm, right wrist, left wrist, right hand and left hand in the past six months. In the same questionnaire, participants report the number of days with neck and upper limb symptoms in the past 6 months (no symptoms, 1–7 days, 8–30 days, 31–90 days, 91–180 days) and the past week (no symptoms, 1 day, 2–3 days, 4–7 days).

5. Number of months without neck and upper limb symptoms in the past six months (0–6 months).

Degree of recovery, pain intensity, number of days with neck and upper limb symptoms and disability will be assessed separately for the neck shoulder region and the arm/wrist/hand region.

#### Secondary outcome variables

1. Physical activity is assessed by means of the validated Short Questionnaire to Access health enhancing physical activity (SQUASH) [30]. The SQUASH questionnaire contains questions about activities in the following four domains: 1) commuting activities (i.e. walking and cycling), 2) activities at work and school, 3) household activities, and 4) leisure time activities (i.e. walking, cycling, gardening, chores, and sports).

2. Body posture and workplace ergonomics during computer work (self reported). Participants rate themselves on the tendency of forward chin movement relative to the trunk in the direction of the computer screen (yes/no), using a document holder (yes/no), location of documents while working with them (right or left from keyboard, behind keyboard, in front of keyboard), keyboard height compared to elbow height (keyboard above elbow height, keyboard at or below elbow height), the tendency to work with raised shoulders (yes/no), keyboard flat on the desk (yes/no), use of mouse (yes/no), left handed (yes/no), hand that controls the mouse (left/right/both), convenient body posture while working with the keyboard and the mouse (yes/no).

3. Body posture and workplace ergonomics during computer work (observed). The following aspects will be observed, using a checklist while the participant is working: position of the participant in relationship to the position of the computer screen (rotation < 10°, rotation ≥ 10°), rotation of keyboard compared to table edge (rotated < 10°, rotated ≥ 10°), rotation of the neck (rotated < 20°, rotated ≥ 20°), height of the computer screen (top of screen far beneath eye height, at eye height, above eye height), viewing distance (shortest distance between the eyes and the computer screen) using a measuring tape, posture of the back while seated (straight/not straight), back supported up to shoulder blades (yes/no), body posture more or less symmetrical (yes/no), support of the elbows while typing (yes/no), ulnar deviation of wrists while typing (yes = ≥ 20°, no = < 20°), lower arm continuously supported (at least 50%) while working with the mouse (yes/no), ulnar wrist deviation while working with the mouse (yes = ≥ 20° or < -5° ; no = < 20° and > -5°).

4. The use of breaks and exercise reminder software and the number of breaks will be assessed by questionnaire.

5. Extrinsic effort and reward (esteem reward, status control and monetary gratification) and need for control, assessed by the short version of the Effort-Reward imbalance questionnaire [31].

6. Decision authority, estimated with the Job Content Questionnaire [32].

7. Phase of behavioural change with regard to physical activity and work style (1. coping with work-related stress, 2. sufficient breaks, and 3. body posture and workplace adjustment), assessed with a questionnaire based on the Trans Theoretical model [33] and the Precaution Adoption Process Model [26].

8. Cardio respiratory fitness, estimated using the validated UKK walk test [34,35]. A short medical questionnaire is used in order to exclude participants from the UKK walk test due to medical reasons.

9. Maximum grip strength, measured using the Jamar hand dynamometer [PGB Active Living, 's-Hertogenbosch, the Netherlands] while the participant is standing with the shoulder in 90° flexion and the arm in full extension.

10. Health care use. At baseline participants were asked whether or not they ever sought medical help for neck and upper limb symptoms. In addition, they were asked to indicate which health care provider(s) they visited. At both follow-up measurements participants were asked again whether or not they sought medical help for neck and upper limb symptoms in the past six months and which health care provider(s) they visited.

### Cost data

Cost effectiveness analyses will be conducted from the employer's perspective. Absenteeism and worker productivity will be based on self-reports using the Health and Performance Questionnaire (HPQ) [36]. Absenteeism will also be assessed by company records.

### Data analysis

Intention-to-treat analyses will be used to estimate the effect of the intervention. Multilevel analyses will be used to investigate differences in recovery and changes in outcome variables between the intervention groups and the control group after six months and one year of follow-up. Multilevel analyses are used in order to adjust for possible dependency between observations from the same company.

Cost-effectiveness ratios will be calculated by dividing the difference between the mean costs by the difference in the mean effects in the primary outcome variables. Bootstrapping will be used to calculate the confidence intervals for the cost-effectiveness ratios. Ratios will be graphically presented on a cost-effectiveness plane. Furthermore, acceptability curves will be calculated for both interventions showing the probability of being cost-effective a specific ceiling ratio.

## Discussion

The RSI@Work study is the first study that investigates the effectiveness of a lifestyle physical activity intervention in addition to a work style intervention on the recovery from neck and upper limb symptoms in computer workers. Initially we intended to study the effectiveness of a third intervention that focused exclusively on physical activity and not on work style. However, during the pilot study the single physical activity intervention was criticized because many participants did no accept the idea that the main cause of their neck and upper limb symptoms was lack of physical activity. Most participants expected bad work place ergonomics to be the primary cause of their neck and upper limb symptoms. Therefore, we decided to study the effectiveness of a combined physical activity and work style intervention and to compare it with the effectiveness of a work style intervention and a control group.

Since research has indicated that people at different stages use different techniques and hold different beliefs about a particular behaviour [37] stage-matched interventions are believed to be more effective than stage unmatched interventions. Evidence supports this expectation with regard to short-term physical activity change but not with regard to long-term physical activity change [38]. As stage-matched interventions are still believed to be promising, we intended to match our participants according to their stage of change with regard to physical activity and work style. However, since our participants can differ in stage of change with regard to two behaviours (work style and physical activity) and are working in different companies, it is nearly impossible to form groups with a sufficient number of participants in each company. Furthermore, work style is a cluster of three behaviours (i.e. 1. body posture and workplace adjustment, 2. sufficient breaks and 3. coping with high workload) which makes a stage-matched intervention not feasible. Therefore, we have decided not to match groups according to stage of change but to benefit from stage differences between participants. Participants in the action phase are encouraged to support participants in the earlier stages to change their behaviour. Although our intervention is not stage-matched, a stage-based approach is used in order to improve work style and increase physical activity. The small group meetings are used to provide participants with stage-based advice with regard to physical activity and/or work style. Although a stage-based approach is nowadays very common in lifestyle interventions, the present study is one of the first to implement a stage-based approach to a work style intervention. To study whether the effectiveness of the intervention differs between participants in different stages of change, stage of change with regard to work style and physical activity will be assessed at T0, T1 and T2.

Although participants are allowed to choose their own physical activities, certain activities seem more promising in reducing neck and upper limb symptoms than others. We expect that physical activities involving the affected body parts will be more effective in reducing neck and upper limb symptoms than physical activities that don't involve the affected body parts. Therefore, participants who meet the guidelines for regular physical activity can still improve their physical activity behaviour by starting physical activities that involve the neck or upper extremities (e.g. swimming, indoor climbing, rowing). Yet, little is known on the effectiveness of specific sports or physical activities in reducing neck and upper limb symptoms. One study found a higher risk of wrist disorders in workers who played tennis [39].

The present study intends to assess the added value of a lifestyle physical activity intervention in addition to a work style intervention that focuses on body posture and workplace adjustment, breaks and coping with high work demands to decrease neck and upper limb symptoms in computer workers. The results of this study will help occupational physicians to decide whether or not to implement a combined approach in the treatment of workers with neck and upper limb symptoms. Furthermore, it will gain insight into the cost-effectiveness of group meetings that aim to improve work style and physical activity in order to reduce neck and upper limb symptoms.

## Competing interests

The author(s) declare that they have no competing interests.

## Authors' contributions

All authors have been involved in the development of the study design. VHH and GAMA participated in the general coordination of the study and read and corrected draft versions of the present manuscript. CMB has been responsible for the data collection and for writing the present manuscript.

## Pre-publication history

The pre-publication history for this paper can be accessed here:



## Figures and Tables

**Table 1:** Guidelines for workplace ergonomics and body posture used in the RSI@Work study

**Topic**	**Advice**
**Workplace ergonomics**	
Seat height	Feet flat on the floor, upper leg completely supported
Seat depth	Lower and upper back completely supported, about 10 cm between back of the knee and front of the seat
Arm rests	Support the elbows (and forearms)
Height of back support	Thickening of back support at belt height
Table height	At same height as armrest while typing, 5 cm above arm rest while reading.
Position of computer screen	Straight in front of worker. Rotation of screen less than 10°. Place screen perpendicularly on window. Distance between screen and eyes dependent on screen dimensions but at least 50 cm.
Computer screen height	Top of the screen at eye height level
Keyboard	Plat on desk, straight in front of screen.
Mouse(pad)	Close to keyboard. Try to alternate the position of the mouse(pad) between right and left of keyboard.
**Body posture**	
Back	Straight. Completely supported by backrest.
Neck	Head rotation less than 20°. Minimal neck flexion/extension (see position of computer screen).
Shoulders	Relaxed (not raised)
Arms	Close to upper body
Hand, wrist, forearm	In straight line. Ulnar deviation between -5° and 20°.
General	Workers were advised to change their posture during the workday.
